# Supplementary material for: SyNDI: synchronous network data integration framework
Source: BMC Bioinformatics. 2018 Nov 6;19:403. doi: 10.1186/s12859-018-2426-5 (PMC6219086; doi:10.1186/s12859-018-2426-5)
Supplement: Supplementary file 5 — Files of the example presented in Additional file 4. This additional file is a zip package that contains all files used in Additional file 4. Detailed descriptions of each of these files are presented in a README file included in this zip package. (GZ 30411 kb) [file 12859_2018_2426_MOESM5_ESM.gz › SyNDITools/Meme2Fimo/meme_4.9.0_4.tar.gz!/meme_4.9.0/etc/ame_template.html]

AME results


The name of the database (file name) that contains the motif.

[
close ]

A name for the motif that is unique in the motif database file.

[
close ]

An alternate name of the motif that may be provided in the motif database file.

[
close ]

Help message goes here.

[
close ]

Help message goes here.

[
close ]

Help message goes here.

[
close ]

Help message goes here.

[
close ]

Help message goes here.

[
close ]

Help message goes here.

[
close ]

# AME (Analysis of Motif Enrichment)

For further information on how to interpret these results or to get a
copy of the MEME software please access
http://meme.nbcr.net.

If you use AME in your research, please cite the following paper:  

Robert McLeay and Timothy L. Bailey,
"Motif Enrichment Analysis: A unified framework and method evaluation",
*BMC Bioinformatics*, **11**:165, 2010, doi:10.1186/1471-2105-11-165.

Enriched Motifs
  |  
Input Files
  |  
Program information


# Javascript is required to view these results!


## Enriched Motifs

| Logo | Database | ID | Name | Split | *p*-value | Corrected *p*-value | Spearman's Rho | Mean-squared-error | m | b |
| --- | --- | --- | --- | --- | --- | --- | --- | --- | --- | --- |

## Input Files

#### Sequences

| Database | Source | Sequence Count |
| --- | --- | --- |
|  |  |  |

#### Motifs

| Database | Source | Motif Count |
| --- | --- | --- |

##### AME version

(Release date: )  
Copyright © Robert McLeay
r.mcleay@imb.uq.edu.au &
Timothy Bailey
t.bailey@imb.uq.edu.au, 2009.

##### Reference

Robert McLeay and Timothy L. Bailey,
"Motif Enrichment Analysis: A unified framework and method evaluation",
*BMC Bioinformatics*, **11**:165, 2010, doi:10.1186/1471-2105-11-165.

##### Command line summary
